# Establishing a genomic database for the medicinal plants in the Brazilian Pharmacopoeia

**DOI:** 10.1186/s13020-021-00484-5

**Published:** 2021-08-05

**Authors:** Guan-Ru Zhou, Bao-Sheng Liao, Qiu-Shi Li, Jiang Xu, Shi-Lin Chen

**Affiliations:** 1 Institute of Pharmacy, Hubei University of Chinese Medicine, Wuhan, 430000 China; 2 Institute of Chinese Materia Medica, China Academy of Chinese Medical Sciences, Beijing, 100700 China

**Keywords:** Genome, Medicinal plants, Brazilian Pharmacopoeia, DNA barcodes, Chloroplast genome, Herbgenomics

## Abstract

**Background:**

Brazil is exceptionally abundant in medicinal plant resources and has a rich ethnopharmacological history. Brazilian Pharmacopoeia (BP) acts as a national standard that regulates drug quality and has six published editions. Recent genomic approaches have led to a resurgence of interest in herbal drugs. The genomic data of plants has been used for pharmaceutical applications, protecting natural resources, and efficiently regulating the market. However, there are few genomic databases specifically for medicinal plants, and the establishment of a database that focuses on the herbs contained in the BP is urgently required.

**Methods:**

The medicinal plant species included in each edition of the BP were analyzed to understand the evolution of the Brazilian herbal drugs. The data of 82 plants in the BP were collected and categorized into four sections: DNA barcodes, super-barcodes, genomes, and sequencing data. A typical web server architecture pattern was used to build the database and website. Furthermore, the cp-Gs of the *Aloe* genus in the database were analyzed as an illustration.

**Results:**

A new database, the Brazilian Pharmacopoeia Genomic Database (BPGD) was constructed and is now publicly accessible. A BLAST server for species identification and sequence searching with the internal transcribed spacer 2 (ITS2), the intergenic region (*psb*A-*trn*H), and the chloroplast genome (cp-G) of Brazilian medicinal plants was also embedded in the BPGD. The database has 753 ITS2 of 76 species, 553 *psb*A*-trn*H and 190 genomes (whole genome and chloroplast genome) of 57 species. In addition, it contains 37 genome sequence data sets of 24 species and 616 transcriptome sequence data sets of 34 species and also includes 187 cp-Gs representing 57 medicinal species in the BP. Analyses of the six cp-Gs of three *Aloe* species identified the variable regions in the cp-Gs. These can be used to identify species and understand the intraspecific relationships.

**Conclusions:**

This study presents the first genomic database of medicinal plants listed in the latest BP. It serves as an efficient platform to obtain and analyze genomic data, accelerate studies regarding Brazilian medicinal plants and facilitate the rational development on their market regulation.

**Supplementary Information:**

The online version contains supplementary material available at 10.1186/s13020-021-00484-5.

## Background

Medicinal plants are sources of phytochemicals that play vital roles in disease prevention and treatment. These sources are inexpensive and readily available and have been used in developed and lesser developed countries [1, 2]. The discovery of Salicin (analgesic and antipyretic) by Rafaele Piria, in 1832, from *Salix alba* [3] is considered a milestone in the development of the global pharmaceutical industry. Since then, medicinal plants have gained considerable importance as sources of bioactive phytochemicals for drug discovery [4, 5]. It has been estimated that approximately 30% of therapeutic drugs are derived from natural resources, particularly plants and microorganisms [6, 7]. In addition, the completion of the Human Genome Project has opened a new chapter in understanding and treating human diseases, initiating and gradually deepening research of herbs at the genomic level [8]. Furthermore, Tu Youyou’s discovery of artemisinin from the plant *Artemisia annua* to treat malaria also demonstrates the potential role of medicinal plants [9].

Herbal medicines in the Latin America came into contact with other medical traditions at the beginning of the sixteenth century, introduced throughout the Conquest and European colonial expansion. Within a dominant sociopolitical framework, the folk herb traditions were fused with other medical cultures and syncretized by religious doctors and other various social workers of certain regional public health systems [10]. During the next three centuries, the historical relationships between native medical traditions in Latin America and the medical cultures of other continents, created a particularly rich ethnomedicinal foundation [11] and continually impacted the national health system of Latin American countries.

Brazil is an ideal country in Latin America that has established itself in public health, emphasizing the application and development of medicinal plants and their derivatives. Brazil has the world’s largest share of biodiversity (15–20%), and most of this biodiversity has not been explored, offering plenty of scope for herbal medicine development [3, 12]. The diverse Brazilian culture contributes mainly to the use of herbal medicines. Besides, approximately 305 ethnic groups, speaking 274 languages [13], have thousands of years of ethnopharmacological history [14]; approximately 1000 plant species have been used as Amazon’s traditional medicine [15, 16]. Additionally, the Portuguese, who colonized Brazil between 1500 and 1822, brought herbs from other parts of the world instead of exploring the native medicinal plants [17]. The traditional herbal medical system with the combined knowledge of the indigenous people, Europeans, and Africans has led to the development of botanical medicines [18]. Consequently, Brazil has become the biggest pharmaceutical market and the only country in Latin America ranked amongst the top pharmaceutical markets worldwide [19]. As the standard publications regulating the quality of drugs, the pharmacopoeias make the quality standards obligatory, ensuring consistency in medicines approved by representatives of specific political units and representing the local progress in related scientific fields [20]. The latest (sixth) edition of BP has revoked all other editions; serves as the core edition of future editions through constant review, seeking to emerge as an international standard. Currently, the Brazilian Pharmacopoeia Commission is an observer for the European and International pharmacopoeias and has a mutual acknowledgement with the Argentine Pharmacopoeia [21]. It will also help guide proposals for the joint development of pharmacopoeias with countries on the South America continent. As a national pharmacopoeia in the Portuguese language, the BP also has an influence on other regions, including Macau, an area that could be a center for medicinal plant development, that integrates Brazilian and Chinese herbal knowledge.

New proteomic and genomic technologies have led to a resurgence of interest in natural products in academia and pharmaceutical organizations [22, 23]. DNA barcode is a short DNA fragment that is different between species [24], and it provides a practical solution for identifying species. In addition, the optimal combination of single-locus barcodes with chloroplast genome (super-barcodes) provides a new method for efficient plant identification [25]. Thus, a database that integrates DNA barcodes and organelle genomes may solve the increasing challenges in plant identification, not just in Brazil, but globally. With the development of sequencing technology and synthetic biology, the transcriptome and genome of plants have been sequenced and used to synthesize the desired compounds by bacterial engineering [26]. For example, ingredients with high medicinal activity, such as artemisinin [27, 28] and paclitaxel [29], have been extracted from medicinal plants. With the help of omics data, the decomposition of biosynthetic pathways of drug compounds has also entered the fast lane [30, 31].

Quality germplasm resources are the key to the generation of omics data. However, various wild resources of plant species have been endangered due to habitat destruction and extensive exploitation and utilization [32–34]. Molecular-marker-assisted breeding based on genomic data can enrich germplasm resources and protect wild resources efficiently [35]. Cultivation of wild medicinal plants has become an inevitable trend of sustainable development [36]. Representative examples of comprehensive omics databases to assist crop breeding are available for rice [37], maize [38], and wheat [39], but none exist for herbs. A database that collects omics data of medicinal plants could accelerate the molecular breeding of elite cultivars.

A genomic database is a warehouse that organizes, stores, and manages a variety of genomic data. There are three public, comprehensive genomic databases available: the National Centre for Biotechnology Information (NCBI), the European Institute of Bioinformatics (EBI), and the DNA Database of Japan (DDBJ). With the rapid increase in the volume and complexity of biological data, these databases play important roles in advancing molecular research [40]. Several studies have published genomic data of medicinal plants; however, problems such as different research team hosts, inconsistent data formats, and unstable web services have brought challenges for the utilization of herb genomic data. Additionally, due to the continuous improvements in genome assembly and species sequencing, the number of genome assemblies within a single species is also increasing [30, 41]. The use of these multiple versions of a genome assembly can be confusing and time-consuming. Therefore, genomic data need to be organized and displayed for further use.

In the present study, the Brazilian Pharmacopoeia Genomic Database (BPGD) [42], a database of the BP medicinal plants containing genetic information, that includes the genome, transcriptome, cp-G, and DNA barcodes, is built. The BPGD will provide a valuable resource for accelerating genome research and the molecular breeding of medicinal plants in Brazil.

## Methods

### Data collection and curation workflow

**Fig. 1:**
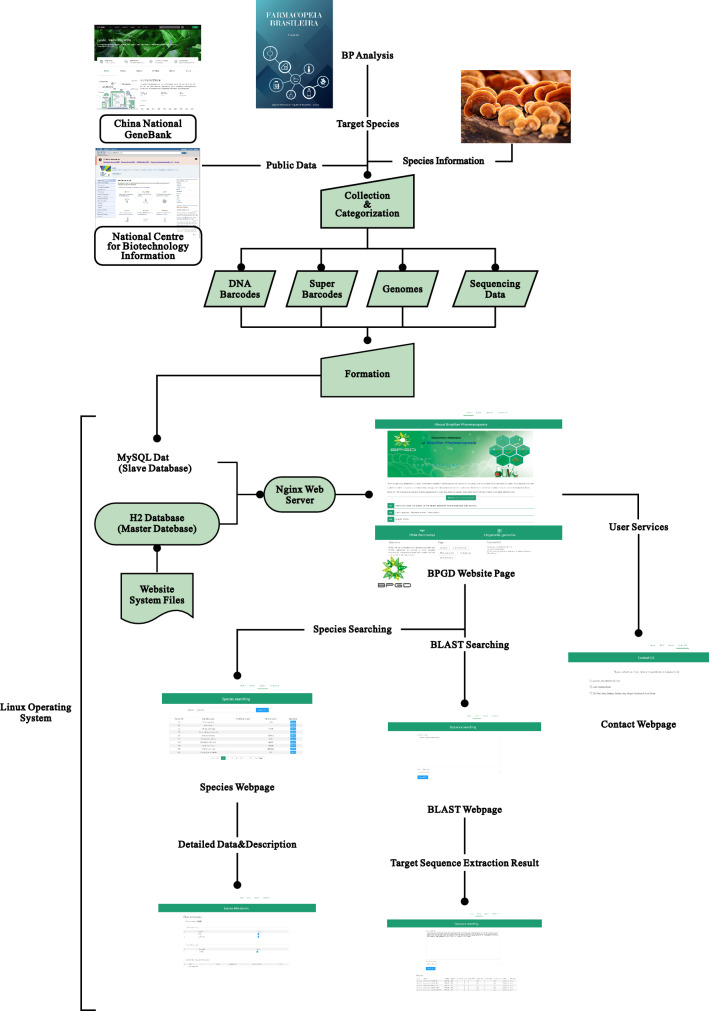
Schematic overview of the BPGD construction pipeline

A schematic overview of the BPGD construction pipeline is shown in Fig. 1. The medicinal plant species of the BP edition were analyzed to understand the changes in the numbers and species of herbal medicines in Brazil. The statistical data of the medicinal plants were collected and categorized from all six editions of the BP. The categories included Latin names, Chinese names, families, relative monographs, occurrence numbers, native or exotic status and other parameters. Details regarding the plant species in the first four editions were collected from a previous work [43], while those in the last two editions were collected and categorized. Latin names that appeared in the BP were adapted according to the monograph description while the native geographical distribution of the plant taxa were adapted according to several modern floras, such as the Flora Reipublicae Popularis Sinicae [44], and the Flora do Brasil 2020 [45].

The genomic data were collected from public databases. The ITS2 and *psb*A-*trn*H sequences available in the NCBI database [46] were linked to the BPGD. The nuclear genomes were downloaded from the NCBI genome database. The majority of raw data were collected from the NCBI Sequence Read Archive (SRA) database [46] and a few from the China National GeneBank database (CNGB) [47]. The uploaded data were categorized into four sections: DNA barcodes, super-barcodes, genomes, and sequencing data sets.

Data of 82 medicinal plants with definite Latin names that were compiled from the plant monographs in the latest (sixth) edition of the BP, were incorporated into the BPGD. Currently, the BPGD (v1.0) has 753 ITS2 of 76 species, 553 *psb*A-*trn*H and 187 cp-Gs of 57 species, the optimal nuclear genomes of three species, and 37 raw genome sequencing data of 24 species, whose assembled nuclear genome data have not been published. In addition, it contains 616 raw transcriptome sequencing data of 34 species.

The cp-Gs were assembled, following the methods described in a previous study [48]. All *Aloe* cp-Gs were aligned using the MAFFT program [49]. The sequence identity in a 50 bp window of the alignment was calculated. The same genes from different cp-Gs were pairwise aligned using Clustal Omega [50], and the pairwise genetic distance was calculated using the EMBOSS program distmat [51] with the default parameters.

### Database implementation

The BPGD was constructed based on the typical web server architecture pattern, LNMP, including the Linux operating system, the Nginx web server with high-performance HTTP and reverse proxy servers, MySQL database server and H2 data management system, and the PHP programming language. A BLAST server was also established to identify the Brazilian medicinal plants based on ITS2, *psb*A-*trn*H, and cp-G.

## Results

### Medicinal plants recorded in the Brazilian Pharmacopoeia (BP)

**Fig. 2:**
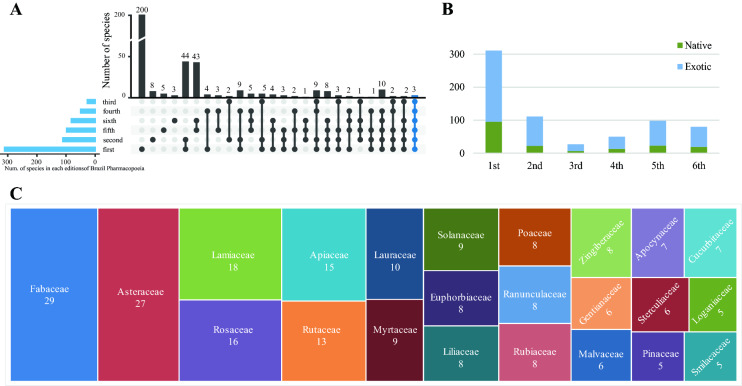
Summary of medicinal species recorded in each edition of the BP. **A** Medicinal species information from different editions of the BP. The dot represents the horizontally aligned edition of the BP, and the line connecting the dots represents the species intersection of the different editions of the BP. **B** Native and exotic species in six editions of the BP. **C** Twenty-two families with the most species recorded in the BP publication history. The area of the rectangle represents the number of species in the family

This study explored the history and development of medicinal plants in the BP (Fig. 2). A total of 200 plants were recorded only in the first edition; 44 in the first and second editions, and 43 in the fifth and sixth editions, which were not included in any other editions. In addition, only three medicinal plant species were recorded in all of the six BP editions and 14 in five editions (Fig. 2A). Additionally, consistent with the earlier editions [21, 52–54], the exotic species were more than native species in the sixth edition (Fig. 2B), indicating the broader applications of non-native herbal drugs in Brazil due to the excellent documentation of quality control [55]. However, a combination of the previous work [21, 52–54] and the data from the last two editions, shows that the number of native plants has risen since the fourth edition of the BP. An analysis of 22 plant families, with most species recorded in the BP history (Fig. 2C), pointed out that eight families alone (Fig. 2C; eight from left) comprised more than 50% of the species in the BP. Thus, the BP analysis confirmed that research on medicinal plants in Brazil is constantly improving.

### Structure of the database

**Fig. 3:**
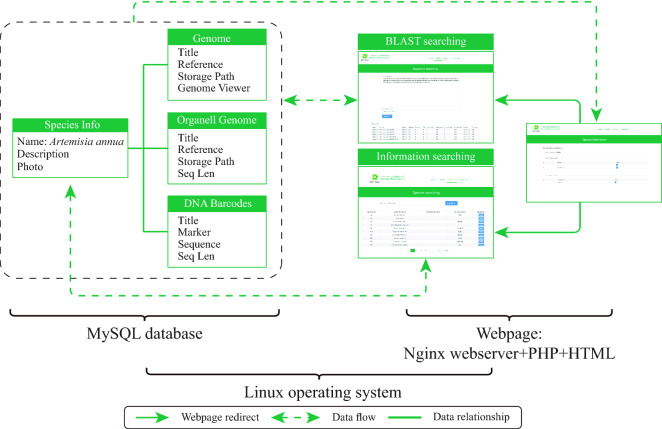
The BPGD structure and the core web pages

The BPGD can be publicly accessed via http://www.bpgenome.com. The database and its web pages are user-friendly (Fig. 3). The BPGD homepage provides the latest information on the compiled BP medicinal plants. The database homepage has a navigation menu consisting of four entries: “Home”, “Blast”, “Species”, and “Contact us”. The homepage also displays a brief introduction and the data collection status. A BLAST tool containing with ITS2, *psb*A-*trn*H, and cp-G sequences provided under the “Blast” entry on the homepage enables search against the genomes and transcriptomes (Additional file 2: Figure S1). Users can view and download the hits from the “Results” page and it shows the species with the highest identity to the query sequence. Through the “Species” page, users can browse the species list and search specific plants using the Latin name keyword. Each plant comes with a “details” tab and the detailed data consist of foursections: DNA barcodes, super barcodes, genomes, and sequencing data sets. Under the “ITS2” and “*psb*A-*trn*H” gateways, twotypes of DNA barcoding nucleotide sequences are available in the Fasta format. Additionally, the optimal nuclear genome data and the links to the original and other assembled versions of the genome can be obtained under the “Genome” gateway.

### Characteristics of the collected cp-Gs in the BPGD

**Fig. 4:**
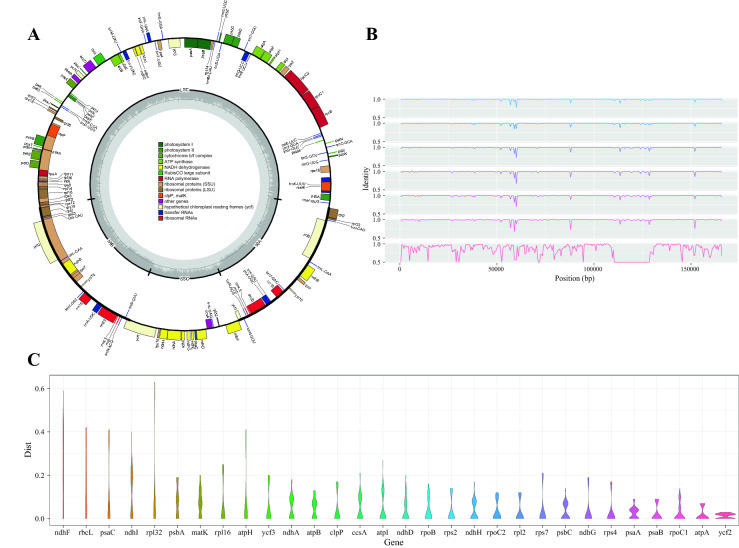
Chloroplast genome analysis. **A** Chloroplast genome of *Aloe vera*. **B** Whole-sequence alignment of three *Aloe* species, using *Belamcanda chinensis* as the outgroup. **C** The pairwise distance for each gene in the chloroplast genome of three *Aloe* species

The chloroplast genome (cp-G) has a suitable length and sufficient variable sites between species, and these sites have been used as super-barcodes to identify plant species [25]. In total, 187 cp-Gs representing 57 medicinal plant species of the BP were collected and stored in the BPGD (Table 1, Additional file 1: Table S1). Babosa (*Aloe vera* L.) is a common species in northeastern Brazil. Its leaves, extracts, and resins have antibacterial, anti-inflammatory, and healing properties and are used to treat liver and stomach diseases [56]. Six cp-Gs of three*Aloe* species (*A*. *vera*, *A*. *maculata*,and *A. barbadensis*; two cp-Gs from each) in the BPGD were analyzed to identify the variable regions in the cp-Gs of these species. The most variable region in the cp-Gs was located in the large single-copy (LSC) region, and the most variable gene was NADH dehydrogenase F (*ndh*F; Fig. 4). The variable region could be used to identify species and understand the intraspecific relationships of the *Aloe* genus.

**Table 1:** Statistics of chloroplast genomes stored in BPGD

Family	Genera	Cp-count
Malvaceae	*Althaea*, *Gossypium*, *Theobroma*	21
Rutaceae	*Citrus*	15
Zingiberaceae	*Curcuma*, *Zingiber*	14
Asteraceae	*Helianthus*, *Matricaria*	13
Fabaceae	*Glycyrrhiza*, *Stryphnodendron*	12
Solanaceae	*Atropa*, *Datura*, *Hyoscyamus*	11
Myrtaceae	*Corymbia*, *Eucalyptus*, *Eugenia*, *Melaleuca*, *Psidium*, *Syzygium*	11
Caprifoliaceae	*Sambucus*, *Valeriana*	9
Apiaceae	*Anethum*, *Centella*, *Coriandrum*, *Foeniculum*	8
Lauraceae	*Cinnamomum*, *Persea*	8
Rosaceae	*Crataegus*, *Prunus*	7
Oleaceae	*Olea*	6
Passifloraceae	*Passiflora*	6
Amaryllidaceae	*Allium*	5
Quillajaceae	*Quillaja*	4
Poaceae	*Cymbopogon*	3
Ranunculaceae	*Hydrastis*	3
Phyllanthaceae	*Phyllanthus*	3
Plantaginaceae	*Plantago*	3
Orchidaceae	*Vanilla*	3
Asphodelaceae	*Aloe*	2
Schisandraceae	*Illicium*	2
Lamiaceae	*Mentha*, *Thymus*	2
Sapindaceae	*Paullinia*	2
Apocynaceae	*Rauvolfia*	2
Polygonaceae	*Rheum*	2
Hippocastanaceae	*Aesculus*	1
Ericaceae	*Arctostaphylos*	1
Sterculiaceae	*Cola*	1
Hamamelidaceae	*Hamamelis*	1
Pedaliaceae	*Harpagophytum*	1
Convolvulaceae	*Operculina*	1
Monimiaceae	*Peumus*	1
Anacardiaceae	*Schinus*	1
Loganiaceae	*Strychnos*	1

## Discussion

Brazil is endowed with unique medicinal plant resources, that play a crucial role in sustaining human health. The history of the BP has been well explained and contains data that help us understand the evolution of medicinal plants. The first edition of the BP, containing a list of plant species that are used in conventional and traditional medicine, is still considered outstanding [57]. This edition also includes 196 monographs of the native herbs that do not exist in any other editions [58], and 42% of medicinal products related to plants [59]. In the early 20th century, the name of traditional Brazilian medicine was generated based on a combination of indigenous knowledge and understanding of the information brought by the Portuguese, Spaniards, and Africans [60]. The Brazilian society transformed severely [61] after the release of the first edition. Sometime in 1940, due to the growth of the global pharmaceutical industry and lack of pharmacological knowledge on the efficacy and toxicity of medicinal plants [62, 63], Brazilians reorganized the pharmaceutical industry to produce drug formulas that utilized imported materials instead of native ones [64, 65]. Later, during World War II, synthetic medicines strongly substituted the traditional medicinal plants [64, 66, 67], which was evident in the subsequent two BP editions. The monographs of plants reduced from 713 in the first edition to 205 in the second edition and 23 in the third edition [52, 68]. Several studies have shown that deforestation affects the natural ecosystem and the devaluation of phytotherapy has threatened medicinal plants and medical knowledge [18, 69, 70]. However, the number of medicinal plants began to grow as the public started favoring phytotherapy. Consequently, the monographs of medicinal plants increased to 17% in the fourth edition [52, 57, 71] and 25% in the fifth edition [58]. Finally, the sixth edition (2019) included a section dedicated to medicinal plants with 149 monographs of plants and plant derivatives. Over nine decades since the first BP was published, the change in the medicinal plants listed in the BP was enormous and the increasing trend of herbal drug use reflected the latest three editions of the BP indicates the recovery of phytotherapy. However, considering the Brazilian biodiversity and the rich phytotherapy history, the patents and medicines based on the BP are few [3]. The combination of traditional herbal knowledge with cutting-edge genomic research methods is of great significance for the understanding and application of medicinal plants in Brazil. Millions of locals would benefit from the use of medicinal plants in health care [72]. The expansion of phytotherapy based on advanced approaches can improve the quality of life of people and contribute to the economic and technological development of society.

Pathogenic microbes, toxic compounds [73, 74], adulterations, and counterfeit drugs [75–78] have raised significant concerns regarding the quality and safety of herbal medicines. The classical analytical procedures provided by the monographs in the BP have projected the difficulties in solving these complex problems related to both exotic and native herbs. Additionally, the natural ecosystem destruction observed in Brazil [67, 79, 80] demands the conservation of Brazilian medicinal plant resources. Because of the global impact of genomics and due to the increasing damage to the domestic ecological environment, the local government and scientists have also made efforts to develop genomics. The Brazilian government began constructing a genetic database in 1984. The Brazilian government organization for Agricultural Research (EMBRAPA) created the National Genetic Resources and Biotechnology Research Center (CENARGEN), aiming at managing the activities related to genetic resources in Brazil [16].

Research on medicinal plants has recently entered the “herbgenomics” era with the development of advanced technologies, especially genomics, transcriptomics, and proteomics. Omics-based analysis has helped us to understand the genetic makeup of herbal medicines revealing their origin, quality, synthesis, safety, and conservation requirements. These technologies will promote the sustainable development of medicinal plants, thereby progressing in human health [81]. The emergence of herbgenomics has enabled systematic research on plant-based medicines from a genetic perspective [8]. Furthermore, molecular-assisted breeding can accelerate the herb breeding progress and protect wild resources with the help of genomics data, for instance, *Panax notoginseng* [82] and *Perilla frutescens* [83].

A publicly accessible database will act as the key hub to efficiently access and interpret the available data sets. To support researchers in handling the rapidly changing data, the development of databases is crucial and challenging. First, genomic data need to be further supplemented. The BPGD (V1.0) collected a portion of the plant species within the latest BP, and the acquisition of more comprehensive genomic data from other medicinal plants in various BP editions will be a challenge, due to difficulties in sample collection and identification, especially for native plant species. Furthermore, the function of the database requires further improvements, and more tools will be developed to meet the requirements of diverse herb studies. An online community will be constructed in order to enrich the data resources and encourage knowledge sharing. Finally, as the database and website are newly developed, there is an uncertainty associated with it as users are only just becoming aware of it. It is our belief that with time as the BPGD becomes more established through development, users will become more familiar with it, engage in the initiative and become willing to use the data and share resources. Several other genomic databases of other well-known pharmacopoeias are currently under construction in our research group, and the completion of the BPGD is the first wave. The BPGD will keep updating data of medicinal plants, and expand to include other data of medicinal plant species in Latin America as a supplement and for possible future applications.

## Conclusions

The database, the BPGD, acts as a bridge that connects the cutting-edge genetic approaches and findings with Brazilian medicinal plants and ethnopharmacy. The BPGD is the first database established that has collected genomic data (DNA barcoding, transcriptome, nuclear genome, and cp-G) of medicinal plants listed in the BP. The database has a ready-to-use data storage function and includes the description, molecular identification, and functions of medicinal plants listed in the BP. The BPGD provides a platform for biological researchers to access the published genetic data quickly and effectively, accelerating research on Brazilian medicinal plants and facilitating the rational development of their market regulation. The BPGD consists of more than 70% of medicinal plant species from the last edition of the BP and will be regularly updated and maintained to provide more comprehensive data and an enhanced user experience.

## Supplementary Information

**Additional file 1: Table S1.** Summary of Cp genomes in the BPGD.

**Additional file 2: Figure S1. **The tutorial of the species identification function of the BPGD.

## Data Availability

The data sets used and/or analyzed during the current study are available from the corresponding author upon reasonable request.

## Abbreviations

BPGD: Brazilian Pharmacopoeia Genomic Database
BLAST: Basic Local Alignment Search Tool
BP: Brazilian Pharmacopoeia
NCBI: National Centre for Biotechnology Information
EBI: European Institute of Bioinformatics
DDBJ: DNA Database of Japan
LNMP: Nginx, PHP, MySQL, phpMyAdmin
HTTP: Hyper Text Transfer Protocol
PHP: Hypertext Preprocessor
ITS: Internal transcribed spacer
NCBI SRA: National Centre for Biotechnology Information, Sequence Read Archive database
CNGB: China National Gene Bank database
LSC: Large single-copy
ndhF: NADH dehydrogenase F
MAFFT: Multiple Alignment using Fast Fourier Transform
EMBOSS: European Molecular Biology Open Software Suite
