# Supplementary material for: Establishing a genomic database for the medicinal plants in the Brazilian Pharmacopoeia
Source: Chin Med. 2021 Aug 5;16:71. doi: 10.1186/s13020-021-00484-5 (PMC8340495; doi:10.1186/s13020-021-00484-5)
Supplement: Supplementary file 1 — Additional file 1: Table S1. Summary of Cp genomes in the BPGD. [file 13020_2021_484_MOESM1_ESM.docx]

**Table S1. Summary of Cp genomes in BPGD.**

| **Species** | **Assembly** | **NCBI accession** | **Source** | **Length** |
| --- | --- | --- | --- | --- |
| *Aesculus hippocastanum* | linear | - | gpdb | - |
| *Allium sativum* | circular | MK335928.1 | NCBI | 153189 |
| *Allium sativum* | circular | KY085913.1 | NCBI | 153118 |
| *Allium sativum* | circular | KY363332.1 | NCBI | 153131 |
| *Allium sativum* | circular | NC_031829.1 | NCBI | 153172 |
| *Allium sativum* | circular | KX683282.1 | NCBI | 153172 |
| *Aloe vera* | circular | NC_035506.1 | NCBI | 152875 |
| *Aloe vera* | circular | KX377524.1 | NCBI | 152875 |
| *Althaea officinalis* | circular | NC_034701.1 | NCBI | 159987 |
| *Althaea officinalis* | circular | KY085914.1 | NCBI | 159987 |
| *Althaea officinalis* | linear | - | gpdb | - |
| *Anethum graveolens* | circular | NC_029470.1 | NCBI | 153356 |
| *Anethum graveolens* | circular | KR011055.1 | NCBI | 153356 |
| *Arctostaphylos uva-ursi* | linear | - | gpdb | - |
| *Atropa belladonna* | circular | NC_004561.1 | NCBI | 156687 |
| *Atropa belladonna* | circular | AJ316582.1 | NCBI | 156687 |
| *Centella asiatica* | circular | MN854377.1 | NCBI | 154771 |
| *Centella asiatica* | linear | - | gpdb | - |
| *Cinnamomum verum* | circular | NC_035236.1 | NCBI | 152766 |
| *Cinnamomum verum* | circular | KY635878.1 | NCBI | 152766 |
| *Citrus aurantium* | circular | NC_052719.1 | NCBI | 160140 |
| *Citrus aurantium* | circular | MT702983.1 | NCBI | 160140 |
| *Citrus aurantium* | circular | MT106672.1 | NCBI | 160140 |
| *Citrus aurantium* | linear | - | gpdb | - |
| *Citrus limon* | circular | KY085897.1 | NCBI | 160101 |
| *Citrus limon* | circular | NC_034690.1 | NCBI | 160101 |
| *Citrus limon* | circular | MT880608.1 | NCBI | 160141 |
| *Citrus limon* | linear | - | gpdb | - |
| *Citrus limon* | linear | - | gpdb | - |
| *Citrus limon* | linear | - | gpdb | - |
| *Citrus sinensis* | linear | DQ864733.1 | NCBI | 160129 |
| *Citrus sinensis* | linear | NC_008334.1 | NCBI | 160129 |
| *Citrus sinensis* | linear | - | gpdb | - |
| *Citrus sinensis* | linear | - | gpdb | - |
| *Citrus sinensis* | linear | - | gpdb | - |
| *Cola nitida* | linear | - | gpdb | - |
| *Coriandrum sativum* | circular | NC_029850.1 | NCBI | 146519 |
| *Coriandrum sativum* | circular | KR002656.1 | NCBI | 146519 |
| *Corymbia citriodora* | linear | KY246394.1 | NCBI | 160277 |
| *Crataegus monogyna* | linear | MT787468.1 | NCBI | 28721 |
| *Crataegus monogyna* | linear | - | gpdb | - |
| *Curcuma longa* | circular | MK621774.1 | NCBI | 159469 |
| *Curcuma longa* | circular | MK965541.1 | NCBI | 162255 |
| *Curcuma longa* | circular | MK919702.1 | NCBI | 159550 |
| *Curcuma longa* | circular | NC_042886.1 | NCBI | 159550 |
| *Curcuma longa* | circular | MN711722.1 | NCBI | 162232 |
| *Curcuma longa* | circular | MT395650.1 | NCBI | 162220 |
| *Curcuma longa* | circular | MK262732.1 | NCBI | 162176 |
| *Curcuma longa* | circular | MK109020.1 | NCBI | 162180 |
| *Curcuma longa* | linear | MG025951.1 | NCBI | 159064 |
| *Curcuma longa* | linear | - | gpdb | - |
| *Cymbopogon citratus* | circular | MK593547.1 | NCBI | 139878 |
| *Cymbopogon citratus* | circular | NC_042144.1 | NCBI | 139708 |
| *Cymbopogon citratus* | circular | MH488954.1 | NCBI | 139708 |
| *Datura stramonium* | circular | MT610897.1 | NCBI | 155884 |
| *Datura stramonium* | circular | MT610896.1 | NCBI | 155884 |
| *Datura stramonium* | circular | JN654342.1 | NCBI | 155871 |
| *Datura stramonium* | circular | JN662489.1 | NCBI | 155940 |
| *Datura stramonium* | circular | NC_018117.1 | NCBI | 155871 |
| *Datura stramonium* | linear | - | gpdb | - |
| *Eucalyptus globulus* | circular | KC180787.1 | NCBI | 160267 |
| *Eucalyptus globulus* | circular | CM024728.1 | NCBI | 160284 |
| *Eugenia uniflora* | circular | NC_027744.1 | NCBI | 158445 |
| *Eugenia uniflora* | circular | KR867678.1 | NCBI | 158445 |
| *Foeniculum vulgare* | circular | NC_029469.1 | NCBI | 153628 |
| *Foeniculum vulgare* | circular | KR011054.1 | NCBI | 153628 |
| *Glycyrrhiza glabra* | circular | NC_024038.1 | NCBI | 127943 |
| *Glycyrrhiza glabra* | circular | KF201590.1 | NCBI | 127943 |
| *Glycyrrhiza glabra* | circular | MT120771.1 | NCBI | 127942 |
| *Glycyrrhiza glabra* | circular | MT120769.1 | NCBI | 127923 |
| *Glycyrrhiza glabra* | circular | MT120767.1 | NCBI | 127952 |
| *Glycyrrhiza glabra* | circular | MG736059.1 | NCBI | 127897 |
| *Glycyrrhiza glabra* | circular | MT120770.1 | NCBI | 127950 |
| *Glycyrrhiza glabra* | circular | MT120768.1 | NCBI | 127985 |
| *Glycyrrhiza glabra* | circular | MT120766.1 | NCBI | 127656 |
| *Glycyrrhiza glabra* | circular | KU891817.1 | NCBI | 127895 |
| *Gossypium hirsutum* | circular | DQ345959.1 | NCBI | 160301 |
| *Gossypium hirsutum* | circular | HQ901197.1 | NCBI | 160265 |
| *Gossypium hirsutum* | circular | HQ901196.1 | NCBI | 160256 |
| *Gossypium hirsutum* | circular | NC_007944.1 | NCBI | 160301 |
| *Hamamelis virginiana* | linear | - | gpdb | - |
| *Harpagophytum procumbens* | linear | - | gpdb | - |
| *Helianthus annuus* | circular | KU315426.1 | NCBI | 151064 |
| *Helianthus annuus* | circular | DQ383815.1 | NCBI | 151104 |
| *Helianthus annuus* | linear | MNCJ02000333.1 | NCBI | 151101 |
| *Helianthus annuus* | circular | NC_007977.1 | NCBI | 151104 |
| *Helianthus annuus* | linear | CM007907.1 | NCBI | 151101 |
| *Helianthus annuus* | circular | MK341452.1 | NCBI | 151100 |
| *Helianthus annuus* | circular | MK341451.1 | NCBI | 151117 |
| *Helianthus annuus* | circular | MK341450.1 | NCBI | 151255 |
| *Helianthus annuus* | circular | MK341449.1 | NCBI | 151096 |
| *Helianthus annuus* | circular | MK341448.1 | NCBI | 151150 |
| *Helianthus annuus* | circular | MN602834.1 | NCBI | 151112 |
| *Helianthus annuus* | circular | MN596419.1 | NCBI | 151151 |
| *Hydrastis canadensis* | circular | MK569495.1 | NCBI | 160089 |
| *Hydrastis canadensis* | circular | NC_034702.1 | NCBI | 160000 |
| *Hydrastis canadensis* | circular | KY085918.1 | NCBI | 160000 |
| *Hyoscyamus niger* | circular | KF248009.1 | NCBI | 155720 |
| *Hyoscyamus niger* | circular | NC_024261.1 | NCBI | 155720 |
| *Hyoscyamus niger* | linear | - | gpdb | - |
| *Illicium verum* | circular | KY085896.1 | NCBI | 143187 |
| *Illicium verum* | circular | NC_034689.1 | NCBI | 143187 |
| *Matricaria chamomilla* | linear | - | gpdb | - |
| *Melaleuca alternifolia* | circular | MN310606.1 | NCBI | 160104 |
| *Mentha piperita* | linear | - | gpdb | - |
| *Olea europaea* | circular | MT182986.1 | NCBI | 155886 |
| *Olea europaea* | circular | MT182985.1 | NCBI | 155531 |
| *Olea europaea* | circular | MT182984.1 | NCBI | 155886 |
| *Olea europaea* | circular | GU931818.1 | NCBI | 155889 |
| *Olea europaea* | circular | NC_013707.2 | NCBI | 155888 |
| *Olea europaea* | circular | GU228899.2 | NCBI | 155888 |
| *Operculina macrocarpa* | circular | KF242502.1 | NCBI | 161475 |
| *Passiflora alata* | circular | MT525869.1 | NCBI | 147773 |
| *Passiflora edulis* | circular | NC_034285.1 | NCBI | 151406 |
| *Passiflora edulis* | circular | KX290855.1 | NCBI | 151406 |
| *Passiflora edulis* | circular | MF807938.1 | NCBI | 151286 |
| *Passiflora edulis* | circular | MN099051.1 | NCBI | 150471 |
| *Passiflora edulis* | circular | MT140635.1 | NCBI | 151316 |
| *Paullinia cupana* | linear | - | gpdb | - |
| *Paullinia cupana* | linear | - | gpdb | - |
| *Persea americana* | circular | KX437771.1 | NCBI | 152723 |
| *Persea americana* | circular | NC_031189.1 | NCBI | 152723 |
| *Persea americana* | linear | MK404308.1 | NCBI | 152732 |
| *Persea americana* | linear | - | gpdb | - |
| *Persea americana* | linear | - | gpdb | - |
| *Persea americana* | linear | - | gpdb | - |
| *Peumus boldus* | linear | - | gpdb | - |
| *Phyllanthus niruri* | linear | - | gpdb | - |
| *Phyllanthus niruri* | linear | - | gpdb | - |
| *Phyllanthus niruri* | linear | - | gpdb | - |
| *Plantago ovata* | circular | MH165324.1 | NCBI | 162116 |
| *Plantago ovata* | circular | NC_041421.1 | NCBI | 149739 |
| *Plantago ovata* | circular | MH205737.1 | NCBI | 149739 |
| *Prunus domestica* | circular | NC_050959.1 | NCBI | 157395 |
| *Prunus domestica* | circular | MT302569.1 | NCBI | 157395 |
| *Prunus domestica* | linear | - | gpdb | - |
| *Prunus domestica* | linear | - | gpdb | - |
| *Prunus domestica* | linear | - | gpdb | - |
| *Psidium guajava* | circular | KY635879.1 | NCBI | 158896 |
| *Psidium guajava* | circular | KX364403.1 | NCBI | 158841 |
| *Psidium guajava* | circular | NC_033355.1 | NCBI | 158841 |
| *Quillaja saponaria* | linear | HQ664570.1 | NCBI | 24557 |
| *Quillaja saponaria* | linear | MH880827.1 | NCBI | 132838 |
| *Quillaja saponaria* | circular | NC_047356.1 | NCBI | 160392 |
| *Quillaja saponaria* | circular | MN709839.1 | NCBI | 160392 |
| *Rauvolfia serpentina* | circular | NC_047244.1 | NCBI | 155102 |
| *Rauvolfia serpentina* | circular | MN746301.1 | NCBI | 155102 |
| *Rheum palmatum* | circular | NC_027728.1 | NCBI | 161541 |
| *Rheum palmatum* | circular | KR816224.1 | NCBI | 161541 |
| *Sambucus nigra* | circular | NC_045061.1 | NCBI | 158205 |
| *Sambucus nigra* | circular | MN524613.1 | NCBI | 158321 |
| *Sambucus nigra* | circular | MN524612.1 | NCBI | 158205 |
| *Sambucus nigra* | circular | MT457821.1 | NCBI | 158102 |
| *Schinus terebinthifolia* | linear | HQ664548.1 | NCBI | 25309 |
| *Strychnos nux-vomica* | linear | - | gpdb | - |
| *Stryphnodendron adstringens* | circular | NC_044627.1 | NCBI | 162169 |
| *Stryphnodendron adstringens* | circular | MN196294.1 | NCBI | 162169 |
| *Syzygium aromaticum* | circular | NC_047249.1 | NCBI | 159370 |
| *Syzygium aromaticum* | circular | MN746306.1 | NCBI | 159370 |
| *Theobroma cacao* | circular | HQ336404.2 | NCBI | 160604 |
| *Theobroma cacao* | linear | JQ228389.1 | NCBI | 160619 |
| *Theobroma cacao* | linear | JQ228387.1 | NCBI | 160619 |
| *Theobroma cacao* | linear | JQ228386.1 | NCBI | 160619 |
| *Theobroma cacao* | linear | JQ228385.1 | NCBI | 160619 |
| *Theobroma cacao* | linear | JQ228384.1 | NCBI | 160619 |
| *Theobroma cacao* | linear | JQ228383.1 | NCBI | 160619 |
| *Theobroma cacao* | linear | JQ228382.1 | NCBI | 160619 |
| *Theobroma cacao* | linear | JQ228381.1 | NCBI | 160619 |
| *Theobroma cacao* | linear | JQ228380.1 | NCBI | 160619 |
| *Theobroma cacao* | linear | JQ228379.1 | NCBI | 160619 |
| *Theobroma cacao* | circular | KY085907.1 | NCBI | 160619 |
| *Theobroma cacao* | circular | NC_014676.2 | NCBI | 160619 |
| *Theobroma cacao* | circular | HQ244500.2 | NCBI | 160619 |
| *Thymus vulgaris* | linear | - | gpdb | - |
| *Valeriana officinalis* | circular | NC_045052.1 | NCBI | 151505 |
| *Valeriana officinalis* | circular | MN524619.1 | NCBI | 151505 |
| *Valeriana officinalis* | linear | - | gpdb | - |
| *Valeriana officinalis* | linear | - | gpdb | - |
| *Valeriana officinalis* | linear | - | gpdb | - |
| *Vanilla planifolia* | circular | KJ566306.1 | NCBI | 148011 |
| *Vanilla planifolia* | circular | NC_026778.1 | NCBI | 148011 |
| *Vanilla planifolia* | circular | MN200375.1 | NCBI | 147714 |
| *Zingiber officinale* | circular | NC_044775.1 | NCBI | 162621 |
| *Zingiber officinale* | circular | MH161428.1 | NCBI | 162621 |
| *Zingiber officinale* | circular | MN736958.1 | NCBI | 161519 |
| *Zingiber officinale* | linear | KM213122.1 | NCBI | 162598 |
