# Supplementary material for: Establishing a genomic database for the medicinal plants in the Brazilian Pharmacopoeia
Source: Chin Med. 2021 Aug 5;16:71. doi: 10.1186/s13020-021-00484-5 (PMC8340495; doi:10.1186/s13020-021-00484-5)

1. Introduction

BPGD (Brazilian Pharmacopoeia Genomic Database) is built by herbgenomics research group, and aims to collect genomic data of medicinal species recorded in Brazilian Pharmacopoeia. A BLAST server for species identification and sequence searching with internal transcribed spacer 2 (ITS2), intergenic region (*psb*A-*trn*H), and chloroplast genome (cp-G) of Brazilian medicinal plants was also embedded in BPGD. The database has 753 ITS2 of 76 species, 553 *psb*A*-trn*H and 190 genomes (whole genome and chloroplast genome) of 57 species, and 37 genome sequence data sets of 24 species and 616 transcriptome sequence data sets of 34 species and also includes 187 cp-Gs representing 57 medicinal species in BP.

2. Species identification

Medicinal species identification function in BPGD can be accessed via http://www.bpgenome.com/search. Steps for DNA Barcoding species identification:

**①** input single sequence which is longer than 50bp

**②** select a database and hit submit button

**③** if there are hits against the database, there will be a list of blast result.


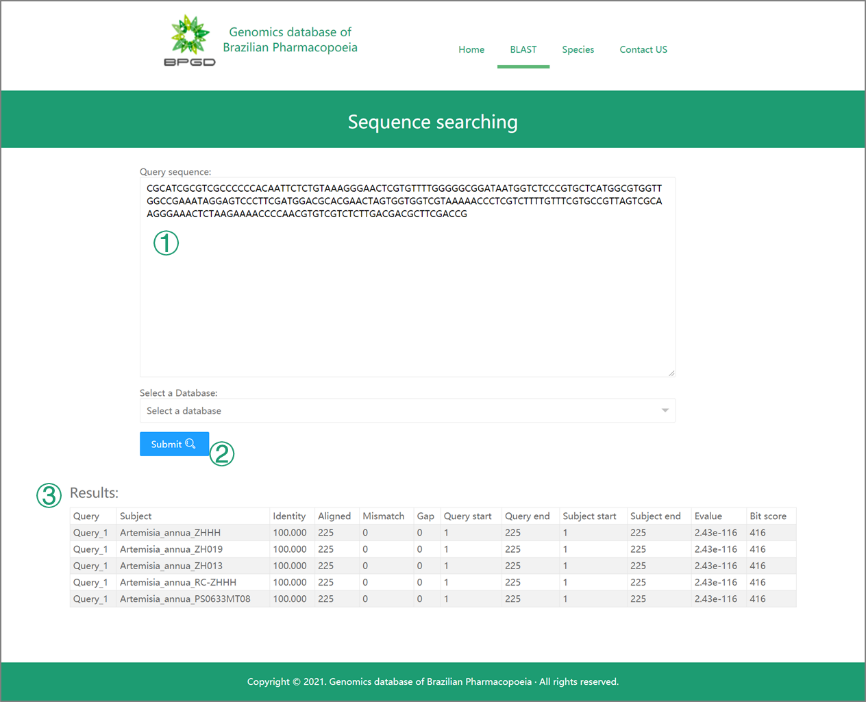

Supplement: Supplementary file 2 — Additional file 2: Figure S1. The tutorial of the species identification function of the BPGD. [file 13020_2021_484_MOESM2_ESM.docx]
